# INVOLVING FAMILY TO IMPROVE COMMUNICATION IN PRIMARY CARE FOR PRIMARY CARE PATIENTS WITH DEMENTIA

**DOI:** 10.1093/geroni/igz038.1328

**Published:** 2019-11-08

**Authors:** Jennifer L Wolff, Sydney Dy, Jennifer Aufill, Diane Echavarria, Cynthia Boyd, David Roth, Jessica Colburn, Danetta Sloan

**Affiliations:** 1 Johns Hopkins University, Maryland, United States; 2 Johns Hopkins Bloomberg School of Public Health, Baltimore, Maryland, United States; 3 Johns Hopkins School of Medicine, Baltimore, Maryland, United States; 4 Johns Hopkins University, Baltimore, Maryland, United States

## Abstract

Family caregivers are at the forefront of managing dementia but are not routinely included in discussions about prognosis and are often poorly prepared to engage in surrogate decision-making. Few interventions target advance care planning in primary care, which is where most persons with dementia are initially diagnosed and medically managed. SHARING CHOICES proactively engages family and support advance care planning in primary care by normalizing advance care planning discussions, clarifying the role of the family during interactions with primary care clinicians, providing ongoing access to a non-clinician trained to lead advance care planning conversations, facilitating registration for the patient portal enable and extending electronic interactions and information access to family caregivers, and providing education and resources about dementia for clinic staff. This presentation will discuss refinement of the SHARING CHOICES protocol and facilitators and challenges of executing a pragmatic trial of this type across two large primary care systems.

